# Georectified polygon database of ground-mounted large-scale solar photovoltaic sites in the United States

**DOI:** 10.1038/s41597-023-02644-8

**Published:** 2023-11-08

**Authors:** K. Sydny Fujita, Zachary H. Ancona, Louisa A. Kramer, Mary Straka, Tandie E. Gautreau, Dana Robson, Chris Garrity, Ben Hoen, Jay E. Diffendorfer

**Affiliations:** 1 Lawrence Berkeley National Laboratory. Energy Technologies Area, Berkeley, CA 94720 USA; 2 United States Geological Survey. Geosciences & Environmental Change Science Center, Denver, CO 80225 USA; 3 University of Colorado Denver. Department of Geography and Environmental Sciences, Denver, CO 80204 USA; 4 United States Geological Survey. Eastern Energy Science Center, Reston, VA 20192 USA

**Keywords:** Energy policy, Databases

## Abstract

Over 4,400 large-scale solar photovoltaic (LSPV) facilities operate in the United States as of December 2021, representing more than 60 gigawatts of electric energy capacity. Of these, over 3,900 are ground-mounted LSPV facilities with capacities of 1 megawatt direct current (MW_dc_) or more. Ground-mounted LSPV installations continue increasing, with more than 400 projects appearing online in 2021 alone; however, a comprehensive, publicly available georectified dataset including spatial footprints of these facilities is lacking. The United States Large-Scale Solar Photovoltaic Database (USPVDB) was developed to fill this gap. Using US Energy Information Administration (EIA) data, locations of 3,699 LSPV facilities were verified using high-resolution aerial imagery, polygons were digitized around panel arrays, and attributes were appended. Quality assurance and control were achieved via team peer review and comparison to other US PV datasets. Data are publicly available via an interactive web application and multiple downloadable formats, including: comma-separated value (CSV), application programming interface (API), and GIS shapefile and GeoJSON.

## Background & Summary

Since 2015, the United States Geological Survey (USGS) and Lawrence Berkeley National Laboratory (LBNL) have collaborated with American Clean Power (ACP) to maintain a highly accurate, detailed, and up-to-date set of georectified data representing US utility-scale wind turbines, the United States Wind Turbine Database (USWTDB)^1,2^; despite the growing deployment of large-scale solar photovoltaic (LSPV), there is no comparable set of data for those facilities across the US. To date, geospatial data describing LSPV facilities tend to be constructed through automated detection of solar panels from satellite images^3^ (a process that faces a number of challenges^4^), include only a subset of facilities^5,6^, represent facilities as single points or circular buffers around centroid points^7,8^, or a combination of the above^9^. This patchwork of data options limits the potential for comprehensive and rigorous analyses.

According to data collected by the US Energy Information Administration (EIA), there are over 4,400 LSPV facilities with capacities of 1 megawatt (direct current) (MW_dc_) or greater in operation in the United States as of December 2021, representing over 60 gigawatts of electric power capacity. Of these projects, over 3,900 are ground-mounted LSPV facilities, i.e., not roof mounted^10^. Ground-mounted LSPV installations continue to grow in number, with over 400 projects coming online in 2021 alone^10^; yet until 2023, there existed no comprehensive, publicly available, and georectified dataset describing the locations and spatial footprints of these facilities. Note that through the remainder of the paper, “MW” refers to direct current (DC or dc) capacity unless otherwise noted. This differs from alternating current (AC or ac) capacity; more details on the differences between AC and DC with regard to LSPV are described in prior solar work, e.g., by LBNL^8^.

Analysts from USGS and LBNL collaborated to develop and release the United States Large-Scale Solar Photovoltaic Database (USPVDB)^11^ building on expertise gained by developing the USWTDB^1^. The USPVDB is currently the most comprehensive set of solar array polygons describing ground-mounted LSPV in the US. The scope of this effort is intentionally limited across several dimensions: (1) residential PV and ground-mounted solar canopies (e.g., over cars in parking lots) are excluded regardless of capacity; (2) facilities with capacities less than or equal to 1 MW are excluded; (3) fourteen solar thermal facilities such as concentrating solar power technologies were excluded; and, (4) regardless of capacity, rooftop PV systems are excluded. While these excluded types of solar power generation might be necessary for a fully comprehensive view of the state of electricity generation in the US, the USPVDB is intended to fill a specific gap in the current analytic landscape of US LSPV to enable analysis of land change aspects of energy production. Moreover, the database represents the overwhelming majority of installed large-scale capacity.

**Fig. 1:**
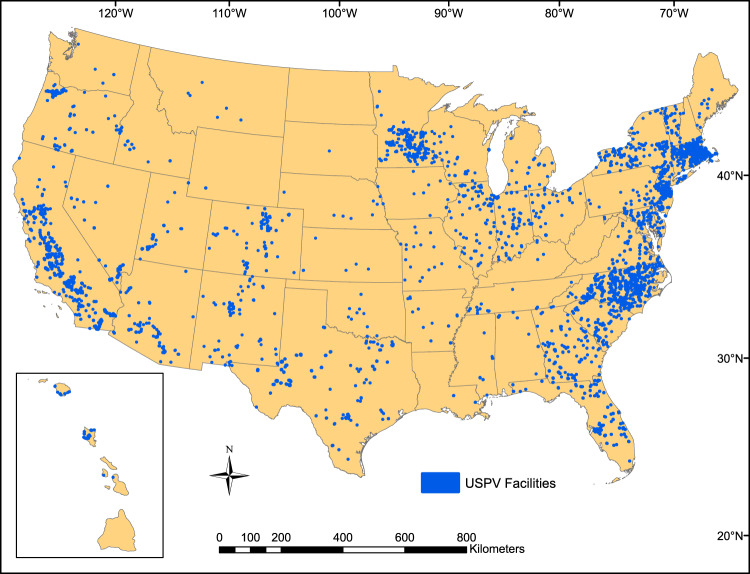
Locations of facilities included in the United States Large-Scale Solar Photovoltaic Database (USPVDB)^11^.

The USPVDB depicts the extent of panel arrays, along with the latitude and longitude of a representative point, for each of 3,699 LSPV facilities. It also provides many attributes for each facility, which are discussed in more detail below. Additional attributes, such as installer name, can be obtained from EIA’s Form 860 database using the USPVDB field “eia_id”, which uniquely links to EIA’s “Plant Code” in their dataset^10^. The USPVDB data are available in a variety of formats, including an interactive web application, comma-separated values (CSV), and polygonal representations (ESRI shapefile, GeoJSON), and are publicly available without cost for use by academic researchers, engineers and developers from PV companies, government agencies, planners, educators, and the general public (Fig. 1).

The USPVDB is intended to enable more accurate analysis of the existing and potential deployment of LSPV as well as the relationships between these projects and other environmental and social land uses and the electricity grid. Examples of research on such topics include: accurate assessments of PV deployment potential; electric grid impacts of renewables; spatial extent of PV arrays^5,6^; PV siting preferences; changes to property values associated with PV^7,12^; and community acceptance of PV^13^.

## Methods

**Fig. 2:**
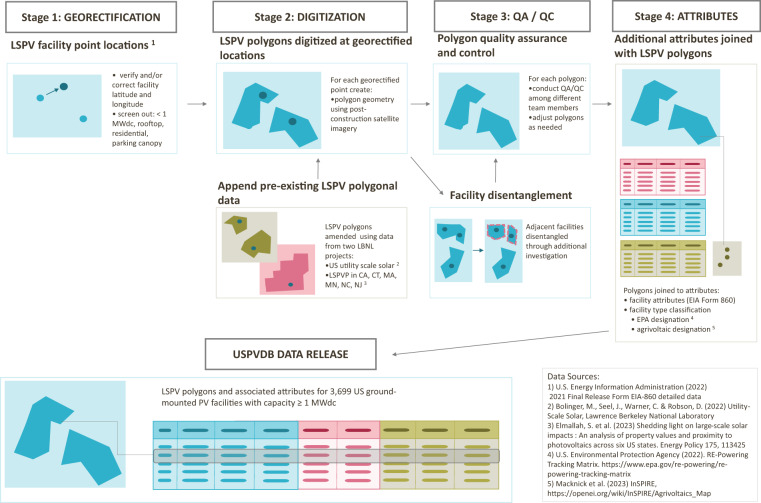
United States Large-Scale Photovoltaic Database (USPVDB) development process.

The USPVDB creation process consisted of four stages, which are summarized here and in Fig. 2, and are described in more detail below:

**Stage 1) Georectifying PV facility coordinates**. Starting from facility latitude and longitude included in EIA data^10^, the locations of LSPV facilities were visually verified using high-resolution aerial imagery;

**Stage 2) Digitizing LSPV array polygons**. Using the georectified PV facility coordinates, polygons were drawn around the extent of panel arrays and inverters. Polygons were drawn manually by USGS and LBNL analysts using ArcGIS software. Polygons from two pre-existing datasets were also revised and incorporated during this stage.

**Stage 3) Quality assurance and quality control (QA/QC), troubleshooting, and in-depth investigations**. Various QA/QC processes were employed to ensure the highest achievable level of accuracy. This included comparing the USPVDB to several other datasets of US LSPV and comparing the included fields against each other statistically.

**Stage 4) Populating facility attributes**. Additional data attributes were appended for each facility, drawing on EIA and several other data sources, as described below.

The stages were completed, to a large extent, in a linear fashion, but there were several iterations of the dataset that required repetition of the steps.

### Stage 1: Georectifying PV facility coordinates

Data from the “2021 final release” of EIA Form 860 were used for solar facility georectifying^10^. These data reflect the near universe of LSPV in operation because EIA reporting is required for facilities with a nameplate capacity of 1 MW or greater if the facility is connected to the local or regional electric grid. EIA Form 860 data include a number of facility attributes (e.g., project name, capacity, operational date, panel type, etc.), which allow for visual and online data confirmation of the facility as needed.

Using EIA Form 860 latitude-longitude coordinates and a suite of imagery services including the ArcGIS DigitalGlobe plugin from Maxar Technologies^14^, Google Maps^15^, and the National Agriculture Imagery Program (NAIP)^16^, each PV facility’s location was examined. DigitalGlobe was the primary source for imagery and others were used sparingly and for verification purposes. Single image tiles were prioritized over mosaics, as this allowed for the collection of accurate image dates, which are recorded in the USPVDB to aid in replicability.

When the EIA coordinates were near a facility, but not on the panel array (or centered between panel arrays in the case of a multi-array facility), the coordinate was updated (i.e., georectified). If no panels were found in proximity to the EIA-provided coordinate, additional steps were taken to confirm the location, including online searching for project information and scanning nearby imagery. Any facilities that constituted the top 1% in terms of distance moved, were further investigated by other members of the team to ensure the georectified point was accurately placed. Other than one instance that was corrected, all “outliers” were confirmed to reflect accurate adjustments of point location approximations during the georectifying process. In total, 2,653 coordinate locations were updated; summary statistics on distances moved can be found in the “Technical Validation” section below.

**Fig. 3:**
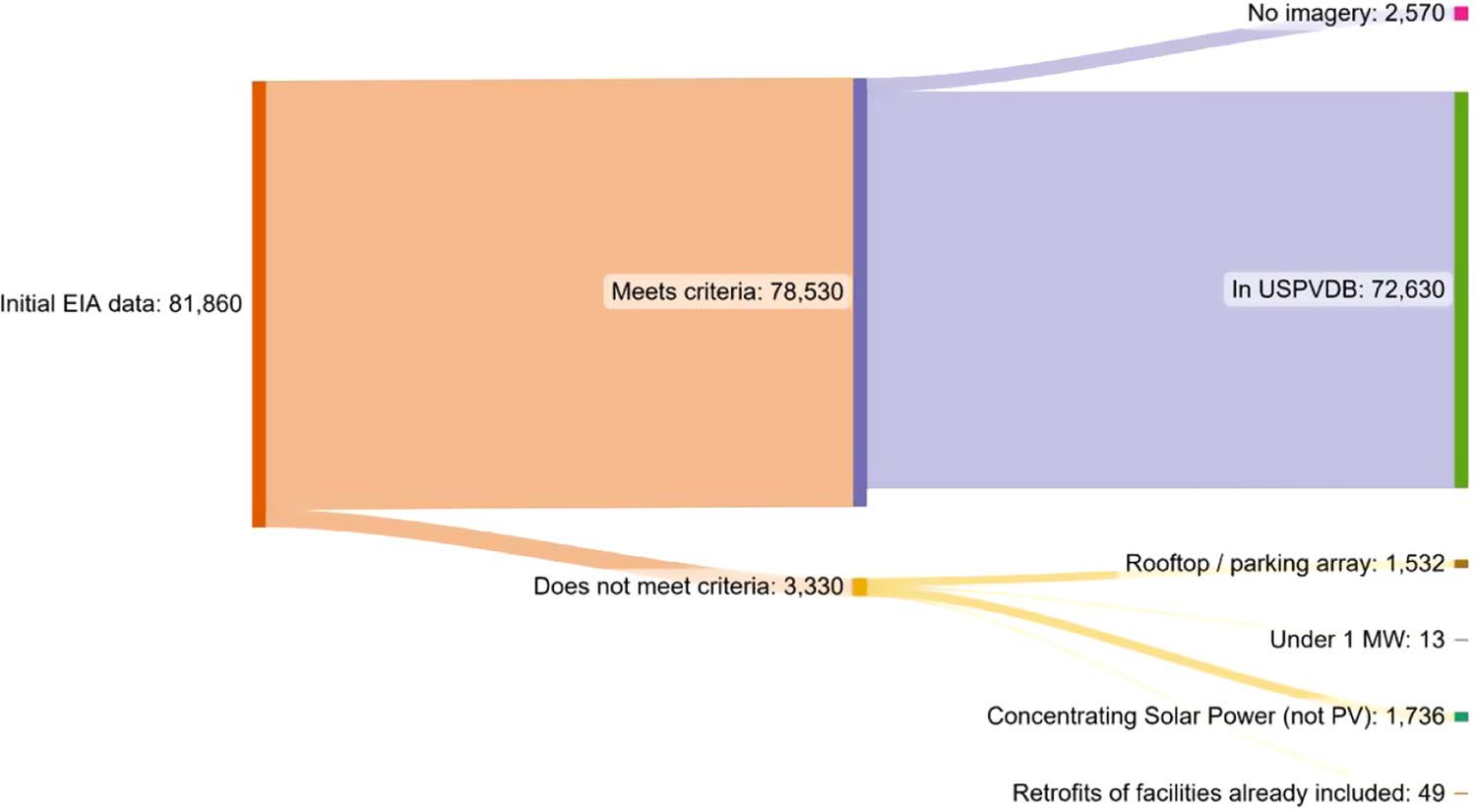
United States Large-Scale Solar Photovoltaic Database (USPVDB) coverage of EIA Form 860 data for relevant facilities (MW_DC_).

Additionally, during this stage, the following criteria were confirmed, or flagged for additional scrutiny: (1) generation technology is PV, (2) panels are ground-mounted but are not parking canopy arrays, and (3) site capacity, estimated from visual inspection, was greater than or equal to 1 MW. Figure 3 depicts facility inclusion in the USPVDB in terms of the total MW capacity of the EIA Form 860 data that: 1) met the criteria and are included in the USPVDB, 2) met the criteria but could not be included due to an absence of high-resolution imagery for the appropriate time period, and 3) did not meet the criteria. A small number of LSPV facilities were found during the preparation of this dataset that were not already included in EIA data. They were not included in this initial release, as they were outside the project scope, but might be included in future releases if additional data can be sourced for them.

### Stage 2: Digitizing LSPV array polygons

This dataset has polygons covering the array area under and around the panels, not the land cover change (i.e., “land transformation”) caused by the facility. This definition of a solar site differs from the full land transformation that may occur during the construction process, and from the full amount of land occupied by the facility inclusive of all of the peripheral infrastructure that may be required (e.g., cleared land, new roads, buildings, transmission, etc.). However, the array area was selected to balance between minimizing the level of effort involved in digitizing each site and maximizing the potential use applications of the final data product. Discussion of more nuanced issues regarding the various methods that researchers have used to map land transformation resulting from PV facility construction can be found in the Usage Notes section below.

DigitalGlobe satellite imagery, provided by Maxar Technologies (MT) was primarily used to determine the extent of solar arrays^14^. This source is regularly updated and convenient to use via an EnhancedView Web Hosting Service (EV-WHS) account and an ArcGIS plug-in to allow the imagery to appear as a base layer. DigitalGlobe data vary in resolution and frequency of imaging across the US. Highly populated areas tend to be more frequently imaged than sparsely populated rural areas, where LSPV are more likely to be sited. Regardless, the clearest image of the facility was selected when digitizing. Because there is always some lag, ranging from several months to several years, between when a facility is fully constructed and the next set of images, more recently constructed solar projects are less likely to be visible in the available satellite imagery. In some cases, where DigitalGlobe imagery was not available (e.g., imagery did not show a facility due to temporal lag or the facility was partially or fully obscured), satellite imagery from the National Agricultural Imagery Program (NAIP) was used^16^.

Solar array polygons were digitized by six team members using ArcGIS software and the imagery described above. Rules were developed to ensure consistent output across team members and address: (1) which facilities to include; (2) how to digitize each facility with consistent accuracy; and, (3) how to save data and track progress. Although facilities were initially inspected during coordinate georectifying, they were reviewed against selection criteria (i.e., > = 1 MW, ground-mounted, no parking lot canopies, residential installations nor concentrating solar, etc.) for a second time when digitizing. Early in the digitizing process, all team members digitized the same set of sites and compared polygons to ensure a shared understanding of the process and across-digitizer reliability. Regular meetings were held throughout the process to discuss difficult to interpret facility images and other digitizing peculiarities and collectively resolve questions. To manage this multi-faceted process, a database was maintained in which the stages of the digitizing process were carefully tracked, facility attributes were recorded, and any notes for use during the QA/QC stage were stored.

When digitizing, the boundary of each polygon was ideally maintained within 5 meters (m) of the outer border of panels, with spacing up to 10 m tolerated. In cases where moderately sized open spaces existed between panel arrays (i.e., 20 m or more), multipart polygons were created. Empty space surrounded by panels (“donuts”) following this criterion were also removed. Provided the accuracy criteria were met, access roads, spacing between rows of panels, and inverters were included. When inverters were distanced from panel rows, they were included by attaching a narrow strip to the rest of the array, ideally no wider than a row of panels. All other site infrastructure was excluded (e.g., driveways not adjacent to arrays, buildings, batteries, etc.). To capture the desired level of detail, digitizing was carried out at a map scale of approximately 1:1000.

Along with newly digitized arrays, polygons from two pre-existing datasets were incorporated and refined during the digitization process. The first was LBNL’s Utility-Scale Solar (USS) dataset, which defines the universe of LSPV facilities with capacities of 5 MW or greater that were operational as of December 2021^5,8^. The second dataset of LSPV facilities with capacities between 1 and 5 MW in six US states that were operational through late 2020 was developed for another LBNL analysis project^12^. These pre-existing polygonal datasets were also based on EIA Form 860 data and followed similar digitizing rules, so they could be easily incorporated into the USPVDB with only minor editing.

### Stage 3: QA/QC, troubleshooting, and in-depth investigations

In the quality assurance and quality control stage, the polygon for each facility was reviewed by at least one team member other than the person who initially created it. In this review, the polygon was inspected to confirm all digitizing rules were followed, and any remaining uncertainties were resolved. In some cases, revisions were made during the QA/QC stage to improve polygon accuracy and consistency.

In some cases, QA/QC involved further investigation (“disentangling”) to confirm site locations and boundaries, particularly in instances where multiple facilities were located in close proximity. These investigations were also conducted by a different team member than the original digitizer and involved extensive web searches for any documents or images that identified the arrays in question. These could include regulatory filings, developer documentation, and news articles. Due to the limits of available resources (e.g., analyst time and aerial image quality), a small number of facilities were unable to be resolved of all uncertainty surrounding exact location and attributes. Such facilities are identified in the USPVDB to allow future data users to tailor the dataset to their analytical needs.

### Stage 4: Populating facility attributes

Once the polygonal dataset was constructed, several additional attributes were merged with the facility geometry to complete the USPVDB. The full list of variables, along with notation of source, is presented in Table 1 and further described in the Data Records section. Many attributes were extracted verbatim from EIA Form 860 data, including facility capacities and other characteristics such as array tilt, panel type, and presence of battery storage. Facilities over 5 MW that are included in the LBNL Utility Scale Solar (USS) Report were validated against this source, and updated to the LBNL values in cases where differences were identified. Aside from the georectification described above, few attribute adjustments were made to EIA data for facilities with capacities of 5 MW or less. Several location attributes were determined based on the polygonal data. They include: state and county that were spatially joined from U.S. Census data^17^ and facility latitude and longitude. The latter were re-computed using the ArcGIS centroid tool and represent the array centroid for single-part polygons, or a point falling on part of an array near the center for multi-part polygons. Finally, facility site type (e.g., greenfield, agrivoltaic) was determined by cross-referencing USPVDB facilities with several pre-existing geospatial datasets, namely the Environmental Protection Agency (EPA) RE-Powering Tracking Matrix (EPA Matrix, or Matrix) and the InSPIRE interactive agrivoltaic map (InSPIRE) maintained by the National Renewable Energy Laboratory (NREL)^18,19^. Those datasets contained geospatial information and other descriptive information that allowed matching to the USPVDB polygons and are described below in more detail. Spatial matching of datasets was performed in the North American 1983 (NAD 83) coordinate system.

**Table 1:** United States Large-Scale Solar Photovoltaic Database (USPVDB) variable sources.

Attribute	Variable Name	EIA	Other	Added or Modified by Analysts
Unique facility identifier	case_id			✓
Facility state	state			✓
Facility county	county			✓
Latitude	latitude			✓
Longitude	longitude			✓
Array area	area_sqm			✓
Multipart polygon indicator	multi_poly			✓
Aerial image date	image_date		MT	
Digitization confidence	dig_conf			✓
EIA identifier	eia_id	✓		
Facility name	fac_name	✓		✓
Year operation began	oper_year	✓		
Regional power authority	pwr_region	✓		
Technology detail	pwr_tech	✓		
Tracking Axis	track_axis	✓		✓
Azimuth	azimuth	✓		✓
Tilt	tilt	✓		✓
Battery storage indicator	batt_stor	✓		
Capacity (megawatts AC)	capac_ac	✓		
Capacity (megawatts DC)	capac_dc	✓		
Facility type category	fac_type		EPA	✓
Agrivoltaic	agrivolt		NREL	✓
Z-Score	z_score			✓

Variable names are as they appear in the USPVDB, which might differ from how they are denoted in the source datasets.

As shown in Table 1, several fields (e.g., “tilt”), were initially obtained from EIA Form 860 data and also modified by the analysts. Where these fields were absent from the original EIA Form 860 data, values were added by the analysts; in other cases, values were updated by the analysts if visual inspection of the site suggested that the original value was inaccurate.

The facilities in the Matrix were matched to the USPVDB polygons based on proximity to the LSPV polygons, facility name, capacity, and date. The public access version of the Matrix does not include latitude-longitude coordinates; these were provided directly to the team by colleagues at the EPA. The Matrix sites are constructed on lands that are “[p]otentially contaminated land include[ing] sites where contamination is suspected but has not been confirmed and sites where contamination has been identified… includ[ing] brownfields, superfund sites, sites subject to corrective action under the Resource Conservation and Recovery Act (RCRA), mining sites, and landfills”^19^. When Matrix sites matched USPVDB polygons, the Matrix site types are included in the USPVDB under “fac_type” (Table 1). When the general “brownfield” category is used in the Matrix, it is recoded as “PCSC” (Previous, current, or suspected contamination) in “fac_type”. An additional five landfills were identified by searching the LSPV facilities for those with “landfill” in their name, which were given the value “landfill_named” in “fac_type”. Finally, USPVDB polygons that did not match the EPA Matrix dataset, were given the value “greenfield” in “fac_type”, and would identify facilities developed on land taken out of other uses, including prior wilderness, desert, urban (e.g., open space in heavily populated areas), rangeland, or agricultural lands.

Agrivoltaic facilities were identified using the InSPIRE database and are denoted within the USPVDB via “agrivolt” (see Table 1)^18^. Agrivoltaic facilities make dual use of land area through agriculturally productive activities conducted between and under PV panels^20–22^. A site is considered agrivoltaic if solar generation and agricultural production are directly integrated within the same land area, such that agricultural activities including crop production, grazing, animal husbandry, or apiaries management occur within the land area occupied by the solar technology infrastructure. Areas providing ecosystem services, such as cover cropping, carbon sequestration, and habitat provision, are also encompassed under the “agrivoltaic” designation. Rooftop PV on a barn or ground-mounted PV adjacent to agricultural land would not be considered an agrivoltaic site. The broad category of agrivoltaic LSPV facilities can additionally be divided into several distinct subtypes: crop production (“crop”), grazing (“grazing”), and ecosystem services (“es”) as identified by the InSPIRE dataset. An individual agrivoltaic facility may provide one or more of these additional values alongside electricity production. These subtypes are provided in the USPVDB field “agrivolt”. If the USPVDB polygon did not match an InSPIRE facility a value of “non-agrivoltaic” is populated in “agrivolt”.

### Other data sources

Maxar Technologies (MT)^14^, Environmental Protection Agency (EPA) – 2022 version^19^, InSPIRE agrivoltaic data mapper managed by the National Renewable Energy Laboratory (NREL) – 2022 version^18^.

## Data Records

Data resulting from this process are available for public use and are provided in multiple formats. These files, including ESRI shapefile, GeoJSON file, CSV, and metadata file (XML) are located at the URL: 10.5066/P9IA3TUS^11^. Additionally, the USPVDB can be accessed through an interactive web viewer where users can interact with the data to explore geographic and temporal patterns in LSPV using advanced filtering and dynamic data symbolization (URL: https://eerscmap.usgs.gov/uspvdb/viewer/)^23^.

The dataset includes the following attribute fields:

**case_id:** unique stable identification number for each facility

**state:** state in which the LSPV facility is located; this is based off the point location to avoid multi-state errors

**county:** county in which the LSPV facility is located, based off the longitude/latitude point location to avoid multi-county errors

**latitude:** latitude of a point representation of the LSPV facility’s location, in decimal degrees, calculated in ArcMap using the calculate geometry tool with the North American 1983 (NAD 83) coordinate system. Representative points are a single point intended to reflect the location of facility panels as accurately as possible; for single-array facilities, they fall in the center of the array, while for multi-part polygons they fall on one of the facility’s arrays.

**longitude:** longitude of a point representation of the LSPV facility’s location, in decimal degrees, calculated in ArcMap using the calculate geometry tool with the North American 1983 (NAD 83) coordinate system. Representative points are a single point intended to reflect the location of facility panels as accurately as possible; for single-array facilities, they fall in the center of the array, while for multi-part polygons they fall on one of the facility’s arrays.

**area_sqm:** area in square meters of the facility array(s), as calculated in the Albers Equal Area Conic projection.

**multi_poly:** facility’s polygon type, which can be:

- **Single** – A single polygon facility is represented by a single part polygon.
- **Multi** – A multi polygon facility is represented by multipart polygon composed of at least two discontinuous polygons that share a single record.

**image_date:** date of image used to visually identify the facility and draw the polygon, as provided in MT’s Digital Globe metadata, format is “YYYYMMDD.”

**dig_conf:** level of confidence in the facility digitization, including description of the reasons for low confidence:

1. multiphase facility or multiple EIA records with identical location. Single polygon used to represent multiple facilities indistinguishable from one another; attributes may not reflect full scope of facilities.
2. multiple polygons created, but EIA records are unclear; attributes may not reflect full scope of facilities.
3. polygon reflects only a part of the facility due to poor image quality; area of polygon may not reflect the full size of array(s).
4. facility polygon created with high confidence. Polygon is expected to represent the full size of the arrays and has a one-to-one connection with the EIA ID.

**eia_id:** unique facility identifier “Plant_Code” from EIA Form 860 data and may be used to associate USPVDB records with other EIA datasets.

**fac_name:** facility name as recorded in EIA Form 860 data.

**oper_year:** year in which the facility started operating, as recorded in EIA Form 860 data.

**pwr_region:** common abbreviation of the relevant regional power authority name (e.g., MISO) for the facility’s location as recorded in EIA Form 860 data.

**pwr_tech:** detail on panel type (e.g., thin film) as recorded in EIA Form 860 data.

**track_axis:** facility’s array axis type (e.g., single axis) as provided by EIA or, in rare cases, determined or adjusted through visual inspection by team members, when appropriate.

**azimuth:** facility’s array azimuth (i.e., east–west orientation in degrees) as provided by EIA or, in rare cases, determined or adjusted through visual inspection by team members, when appropriate.

**tilt:** tilt angle of the facility’s panels (i.e., angle of panels from horizontal in degrees) as provided by EIA or, in rare cases, determined or adjusted through visual inspection by team members, when appropriate.

**batt_stor:** indicator of the presence of battery storage at the facility as recorded in EIA Form 860 data.

**capac_ac:** facility capacity in MW AC as recorded in EIA Form 860 data.

**capac_dc:** facility capacity in MW DC as recorded in EIA Form 860 data. In two cases, these were infilled using the average DC to AC ratio of the full sample.

**fac_type:** general categorization of the facility type, with field values drawn from EPA’s RE-Powering Matrix^19^. Previous, current, or suspected contamination (PCSC) sites have been designated by the EPA as either general “brownfield” sites or given more specific designation regarding the potential for contamination. The land underlying PCSC facilities is *suspected* to be previously or currently contaminated through use for, e.g., mining, military purposes, chemical processing, etc. Such lands may be limited in potential uses due to health and safety constraints. Values of “fac_type” are as follows:

- **RCRA:** Resource Conservation and Recovery Act (RCRA) sites as designated by the EPA Matrix dataset. These are a specific category of commercial, industrial and federal facilities that treat, store or dispose of hazardous wastes and that require cleanup under the RCRA Hazardous Waste Corrective Action Program.
- **superfund:** inactive or abandoned contaminated facilities or locations where there is an active release or threatened release into the environment of hazardous substances that have been dumped, discharged, emitted or otherwise improperly managed. These sites may include manufacturing and industrial facilities, processing plants, landfills and mining sites, among others and are taken directly from the EPA Matrix dataset.
- **AML:** Abandoned Mine Land (AML) sites as designated by the EPA Matrix dataset. These sites include abandoned hardrock mines and mineral processing sites listed in the Superfund Enterprise Management System (SEMS) at this time.
- **landfill:** sites designated as landfills in EPA’s RE-Powering Matrix.
- **landfill named:** sites designated as landfills when the facility name includes the word “landfill”, but the site was not identified as a landfill in the EPA Matrix dataset. It is possible that these sites have been sufficiently cleaned or were never contaminated to the point of meeting the PCSC designation; they are thus distinguished from EPA designated landfill sites.
- **PCSC:** sites appearing in the EPA Matrix dataset as simply “brownfield” or “other” and, therefore, not designated as one of the above site types.
- **greenfield -** Greenfield facilities encompass the remainder of LSPV facilities that do not fall into one of the above-named categories. Greenfield sites represent the majority of LSPV facilities and occupy land that may have previously been wildland, urbanized, cultivated, or reclaimed.

**agrivolt:** agrivoltaic facilities, as denoted in the InSPIRE dataset. These sites make use of the land between panel rows, under arrays, and surrounding arrays for agricultural purposed (i.e., crop production or grazing) and/or ecosystem services (e.g., pollinator habitat). Four non-mutually exclusive values distinguish agrivoltaic types:

- **crop:** site used for agricultural crops
- **es**: site includes ecosystems services, including native pollinator habitat
- **grazing:** site used for livestock grazing
- **non-agrivoltaic:** not an agrivoltaic site

**z_score:** a measure, in units of standard deviation, of how much a facility’s capacity density estimate differs from the mean across the full sample. Larger absolute values of scores indicate potential error in the digitizing or source attributes. This field was calculated by first converting the area_sqm to hectares (ha) by dividing by 10,000, then estimating capacity density (MW/ha) by dividing capacity (MWdc) by area (ha). Z-scores were then estimated as follows where *D*_*i*_ is the capacity density, $\underline{D}$ is the sample mean and *σ*_*D*_ is the standard deviation of the sample mean:

$${z}_{i}=\frac{{D}_{i}-\underline{D}}{{\sigma }_{D}}$$

Any missing values in the GIS files are left blank for text fields and replaced with −9999 for numeric fields. In the CSV, missing text fields are also left blank and numeric fields are left containing null values.

## Technical Validation

As noted above, the USPVDB data underwent rigorous QC and validation including the use of high-resolution aerial imagery to validate LSPV footprints, and an internal peer review of polygons by at least one additional team member. Additionally, USPVDB data were compared to other US PV datasets. While no other datasets were identified that rival the USPVDB in terms of both geographic coverage and detail of facility attributes, these pre-existing datasets provide useful points of comparison to better understand the value of the USPVDB and its level of precision. Both internal QC/QA results and those compared to external datasets are described below.

### Internal validation and quality assurance

Human error was minimized through the use of multiple internal peer review processes as discussed above; details of those processes are here quantified.

As noted above, during the initial georectifying, 2,653 point representations of facility locations were moved from their initial coordinates as collected from EIA data. The mean distance moved was 0.5 km, while the median was 0.1 km, indicating most adjustments were small. 10% were moved 1.1 km or more. The coordinates that were moved were located in urbanized areas not in proximity to any PV arrays. It is believed, in these instances, the EIA location was an office address of the PV facility owner, rather than the facility address.

Additionally, the team was not able to observe 183 facilities via aerial imagery (see Fig. 3). For those, even though a point location was provided by EIA, no polygon was created, and they are excluded from the dataset. Almost all of these facilities became operational between 2018 and present, and they represent only 3% of the total available data from EIA. Although not normal, delays in areal imagery provision do exist, which is likely the case here. These sites may be added as imagery becomes available.

**Fig. 4:**
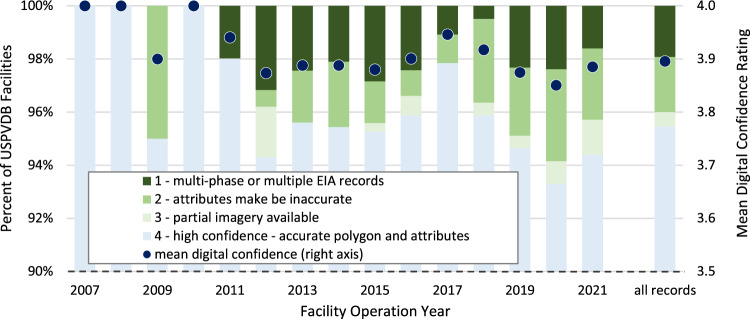
Digitizing confidence score (dig_conf), by operational year (January 2007 - December 2021). Note truncated y-axes.

For all facilities in the dataset (i.e., those with polygons), a confidence level (“dig_conf”) is provided to capture the quality of the aerial imagery and related attributes. The distribution of these ratings is provided by operational year and the full dataset in Fig. 4 (note truncated y-axes ranges). Overall, we are highly confident (i.e., dig_conf = 4) of 3,531 (95.5%) of the LSPV facility polygons. We are moderately confident in the digitizing accuracy of 20 (0.5%) facilities (dig_conf = 3), for which it is believed the location is correct, but image quality prevented ensuring that the entirety of the PV array area was included in the polygon. There is lower confidence in the remaining polygons. For 77 of them (2.1%), a best attempt was made to disentangle multiple nearby facilities, appropriate attribution of each facility could not be conclusively confirmed (dig_conf = 2). For 71 others (1.9%), a single polygon was created to represent either multiple phases of a facility or multiple adjacent facilities (dig_conf = 1); certain attributes in the records for these combined facilities may not accurately reflect, e.g., the total capacity.

### Comparison to other external datasets

Comparisons to five other PV datasets were performed to broadly characterize: how well the USPVDB captured existing facilities; the quality of USPVDB polygons; and, if anomalous (i.e., likely incorrect) records existed. The comparison datasets were constructed in a variety of ways, using different data sources, and for multiple purposes, as described below. This allowed us to compare our methods to a variety of others:

- Dunnett *et al*. extracted global (including the US) PV points and polygons from Open Street Maps (OSM) tagged as solar, which they improved using machine learning (ML) and satellite imagery, to roughly estimate global installed capacity. They published only the points but provided facility area and capacity for each point.^9^;
- Kruitwagen *et al*. used ML to draw PV polygons globally, with the assistance of manually drawn non-US training data, to model global installed capacity^3^;
- Ignizio and Carr manually digitized LSPV surface land transformation associated with 33 facilities in CO and NM, but not from the Colorado River Basin^24^;
- Carr *et al*. manually digitized over 1,000 solar facilities installed through 2015 exclusively using NAIP aerial imagery and EIA Form 860 data for the Bureau of Land Management^25^;
- Shook manually digitized polygons from surface land transformation associated with 12 PV facilities in the Colorado River Basin^26^.

### Data completeness

As noted above, the main source of data for the USPVDB (EIA Form 860), is not perfectly complete, but is expected to contain the overwhelming majority of facilities. Operators of LSPV facilities are required to submit EIA Form 860 if (1) total generator capacity is 1 MW or greater, (2) the facility is connected to the local or regional power grid, and (3) the facility is scheduled for initial operation within 5 years.

A small number of facilities were found while georectifying and drawing polygons that are not in the EIA dataset – they may be added in the future. To examine how well the USPVDB represents the actual number of PV facilities in the US, USPVDB facilities were compared to Dunnett *et al*.’s OSM point data^9^. For the analysis, it was assumed the OSM dataset served as an independent, national-scale mapping effort of PV. Dunnett *et al*.’s points were verified using imagery from March 2020 and prior. Therefore, in this comparison, only USPVDB polygons for projects that began operation in 2020 or earlier were considered. The USPVDB includes 3,348 polygons installed between 1984 and 2020, while Dunnett *et al*. contains 1,082. When joined using a 3,000-meter buffer, there were 1,107 matches between USPVDB polygons to the OSM points, resulting from many-to-one relationships when multiple polygons or points were within 3,000 meters.

The comparison reveals that while the OSM data do not capture approximately 2,200 facilities included in the USPVDB, the USPVDB captures the overwhelming majority of the OSM points. Of the 1,082 OSM points, 928 (86%) matched existing polygons in the USPVDB, 101 (9%) did not meet the criteria for inclusion (e.g., rooftop PV, or <1 MW), and 53 (5%) met USPVDB criteria, but were not yet in the USPVDB. Similar analyses were attempted with data from Kruitwagen *et al*.^3^; however, there were too many instances when the ML algorithm drew a single polygon around two different, but closely spaced, facilities, or it split a single facility into multiple polygons. This made the dataset unfit for a meaningful comparison to the USPVDB.

### Digitizing quality and level of detail

**Fig. 5:**
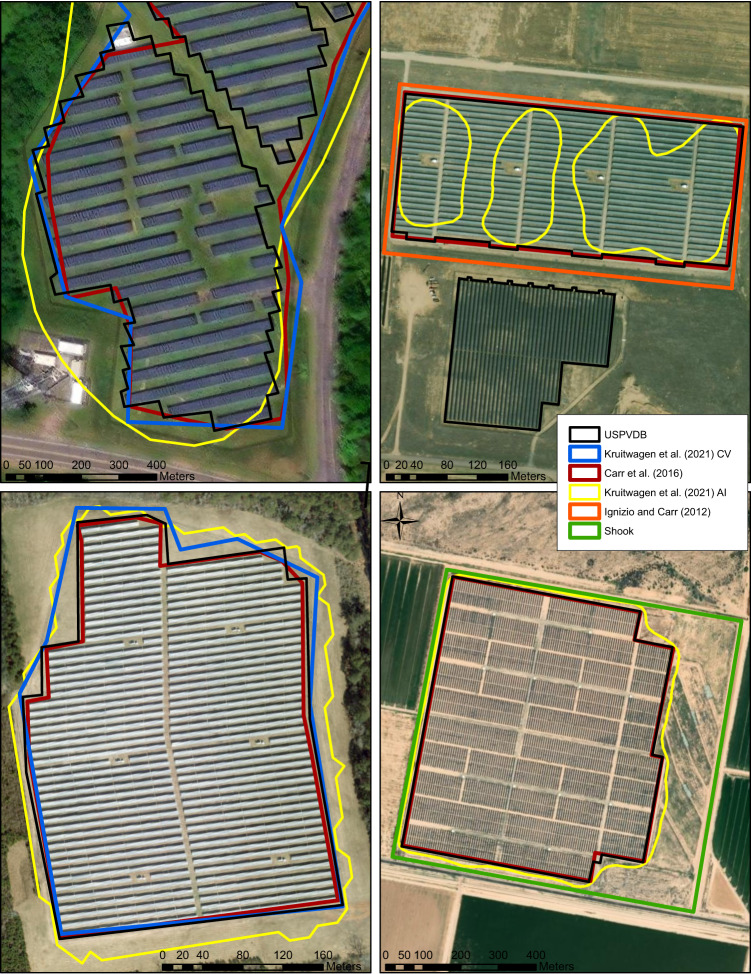
Polygons generated by different mapping efforts at four PV facilities in the US. Kruitwagen *et al*.’s polygons were generated by machine learning. Ignizio and Carr, and Shook digitized land transformation, while the other efforts focused on panel area.

Visual examination of polygons at locations where many research efforts mapped the same facility, indicates the approach used to create the USPVDB polygons is similar to previous manual digitizing efforts (Fig. 5) and ranges from marginally to considerably better^24,25^. Polygons derived through ML, such as by Kruitwagen *et al*. were somewhat less accurate, but were still of reasonable quality for generalized outlines and facility locations considering the global scope of the mapping effort^3^.

Statistical comparisons were also made to provide support for the visual examinations. This was done on an overlapping set of polygons. To prepare, each dataset was filtered to: exclude non-PV technologies, such as concentrated solar and mixed technologies; exclude points outside the US from global datasets; and, remove data with zeros or null values for area. Polygon areas were skewed in nearly all datasets, so non-parametric approaches were applied in two analyses.

**Fig. 6:**
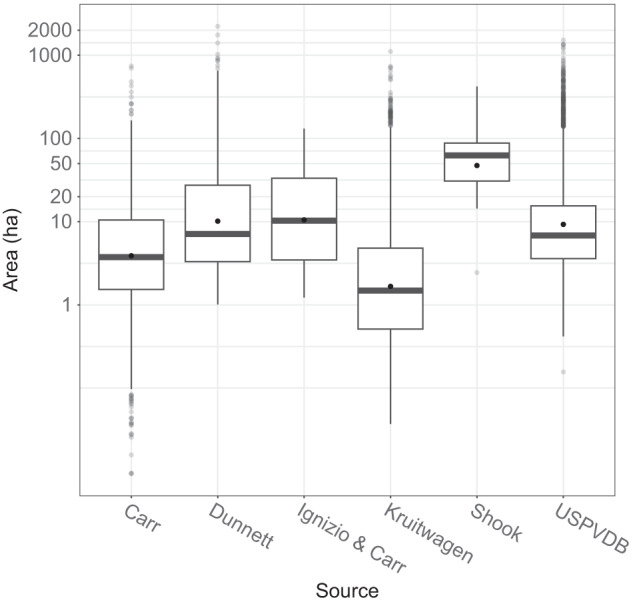
Boxplots of the area (hectares) of each polygon in 6 different PV mapping efforts. The black dots in the boxes represent mean values.

First, Kruskal-Wallis tests were conducted to evaluate the statistical significance of differences between median polygon areas across the datasets. These tests were conducted between pairs of polygons within each dataset, resulting in 15 total comparisons. All pairs of medians differed significantly (p < 0.05) except for the following: Dunnett *et al*. vs. Ignizio and Carr; Dunnett *et al*. vs. Shook; Ignizio and Carr vs. Shook; Ignizio and Carr vs. USPVDB; and, Shook vs. USPDVB. Distributions of polygon areas across each of the 6 datasets are shown in Fig. 6. The smaller datasets (Ignizio and Carr, and Shook) do not include the larger, but somewhat rare, facilities. The USPVDB, on the other hand, includes a greater number of large facilities, likely because of its comprehensiveness and a trend toward larger average facilities in recent years.

In the second analysis, unique pairs of polygons are compared between USPVDB and the other datasets that spatially overlap as detected using spatial joins in ESRI ArcGIS. Due to disentanglement issues associated with the EIA data, as described in the workflow above, only facilities digitized with a single polygon in the USPVDB were compared to others. For this analysis, data from the two surface land transformation digitizing efforts were combined (Ignizio and Carr; and, Shook), because they did not overlap geographically (these are referred to as “Land Transformation” in the table and figures below)^24,26^. Comparisons were limited to 1:1 spatial joins, meaning instances were discarded where other datasets joined more than one polygon to the single USPVDB polygon. This was done because, as noted above, there were numerous cases where the ML algorithms grouped two smaller facilities into a single facility, and instances where a single facility was split into multiple polygons (e.g., upper right panel, Fig. 4). In these cases, the count of the number of spatial joins were always >1 so they were removed from the spatially matched data prior to analysis. Because the Dunnett *et al*. dataset only contained points, they were not used for this matched-polygon analysis.

**Fig. 7:**
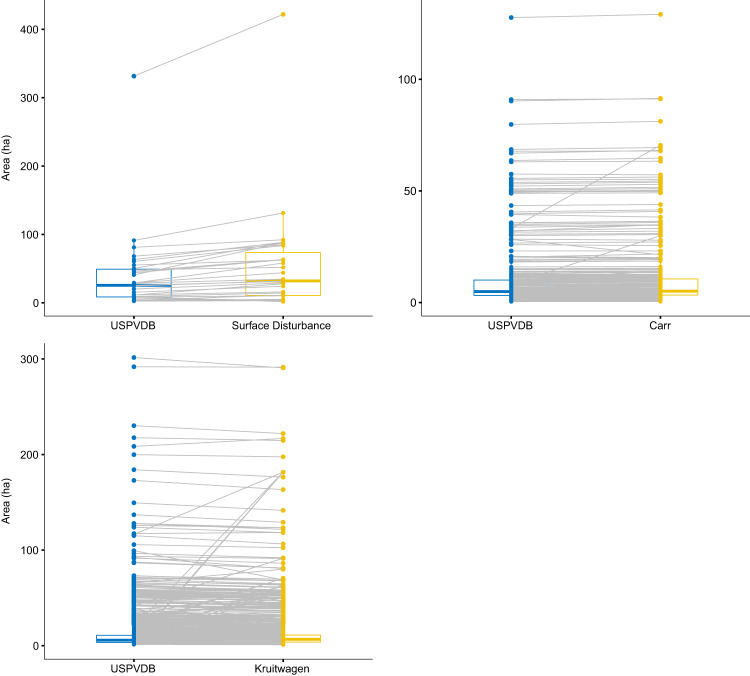
Box plots and spaghetti diagrams of the area of digitized polygons for PV facilities shared across validation datasets and United States Large-Scale Solar Photovoltaic Database (USPVDB). “Land Transformation” represents combined polygons from Ignizio & Carr and Shook. Lines link the same facility across the two datasets.

The pairs were compared using a Wilcoxon signed rank test. They suggest statistically significant, but small, magnitude differences in the mapped areas of PV relative to previous mapping efforts (Table 2 and Fig. 7). Polygons digitized by Carr *et al*. were slightly smaller than those in the USPVDB, as were the ML-based from Kruitwagen *et al*.^3,25^. As anticipated, the combined land transformation dataset had larger areas than the USPVDB, with a median area approximately 25% larger than the paneled area^24,26^. These differences are similar to those reported in a separate analysis by Ong *et al*., where the mean percent difference in area between “direct area” (panels only) and total area (panels plus surface disturbance) across 111 facilities is approximately 38%^27^.

**Table 2:** Differences in PV dataset facility area estimates using Wilcoxon signed-rank test.

Other Datasets	n	Comparison Set			USPVDB			Difference	
		Median	Mean	SD	Median	Mean	SD	V	*p*-value
Carr *et al*.	316	4.95	11.5	16.9	5.11	11.9	17.4	6,470	0.000
Kruitwagen *et al*.	1,075	5.77	13.1	25.9	6.62	14.2	27.2	320,188	0.002
Land transformation	31	32	54.1	76.7	25.5	39.7	59.7	473	0.000

### Anomalous records

The USPVDB largely relies on data from EIA for both spatial and attribute information. Unlike the US Wind Turbine Database^1^, where the collaboration with ACP ensures high quality turbine attributes linked to specific facilities and turbines, ancillary information is not yet available to validate EIA information.

**Fig. 8:**
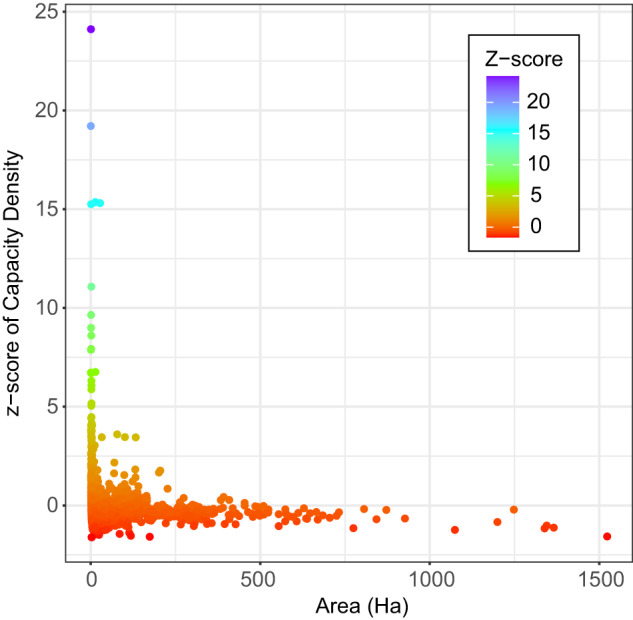
Scatterplot of the z-score of capacity density (MWdc) vs Area for the United States Large-Scale Solar Photovoltaic Database (USPVDB) facilities. Points are color-scaled by the z-score.

To identify potentially anomalous records in the dataset, at least in terms of installed capacity and polygon area, the z-score was examined. As noted above, z-scores were calculated for capacity density (MWdc/ha) across the full dataset. Records with extremely high or low scores may have incorrect values for capacity or polygon area, or both. Of the 3,699 total facilities, 43 have z-scores larger than 3, while the lowest z-score is only −1.61. Large positive z-scores suggest power density is overestimated. This would occur when the reported capacity is larger than the actual, or because the polygon area is smaller. Figure 8 indicates most facilities with high z-scores were small. This suggests that high z-scores occur when the complete facility was not identified, resulting in a polygon that underrepresents a larger facility. This might occur if a ground-mounted array is adjacent to a rooftop or parking canopy PV installation. The latter two are excluded from the USPVDB. For these facilities, the MW capacity listed in EIA records may reflect the entire facility, not just the ground-mounted solar arrays, resulting in a larger MW capacity listed for a smaller ground-mounted facility. Negative z-scores are less concerning because of their small relative magnitude, but they might indicate the reported capacity is smaller than actual, or the polygon contains parts of another, nearby, facility.

## Usage Notes

Users of the USPVDB should consider a number of caveats.

The intent of the USPVDB is to capture large-scale PV facilities composed of ground-mounted arrays with capacities of 1 MW_dc_ or greater. Every effort was made to ensure that solar technologies of other types, including rooftop and parking canopy PV, smaller ground-mounted systems, and residential PV are excluded. Several concentrating solar-thermal power (CSP) facilities were identified over the course of the work but were excluded as well.

The dataset contains nearly the entire universe of PV systems meeting these criteria installed through December 2021. Therefore, it does not contain facilities that have come online since January 1, 2022. Further, because high resolution aerial imagery was either not available post-construction, or the available imagery was not of sufficient quality to identify the facility footprint (e.g., too much cloud cover or image strips only covering a portion of the facility) a small portion of facilities that became operational prior to 2022 were not digitized.

While every attempt was made to distinguish between facilities in close proximity to one another (and between different stages of large facilities), there remain instances where sufficient information could not be found to make this determination. In such instances when arrays of multiple facilities are present but cannot be confidently identified, polygons are included for them with this uncertainty denoted in the dataset (dig_conf = 1).

Anomalous records, in terms of power density, can be identified using the z-score field. Facilities with high z-scores are included in this release of the USPVDB, with plans to conduct additional QA/QC and validation to address these issues in future iterations of the database. However, such extreme values should not be used in analyses of power density as the record likely has an error. Given that all outliers were positive, including the anomalous records in analyses of capacity density would likely overestimate fleetwide values.

Users should be aware that although great efforts were made to validate the locations and attributes of LSPV facilities through inspection of aerial imagery, none of the facilities are field verified.

The polygons were digitized around PV arrays and do not reflect the total land transformation associated with solar PV energy. Users should recognize these polygons will underestimate land transformation and energy density as compared to sources that define solar sites more broadly^27,28^.

If updates to the USPVDB occur, it is expected they will incorporate additional facilities that have come online in the previous year and may also include older facilities that are currently excluded due to the lack of appropriate satellite imagery. Additionally, updates to the USPVDB may include the addition of further attribute fields, such as a formalized confidence indicator developed from the z-score.

## Data Availability

No custom code was used in the development of this dataset.
